# Self-reported use of anti-malarial drugs and health facility management of malaria in Ghana

**DOI:** 10.1186/1475-2875-6-85

**Published:** 2007-07-02

**Authors:** Kwame O Buabeng, Mahama Duwiejua, Alex NO Dodoo, Lloyd K Matowe, Hannes Enlund

**Affiliations:** 1 Department of Social Pharmacy, University of Kuopio, Finland; 2 Department of Clinical & Social Pharmacy, Faculty of Pharmacy, Kwame Nkrumah University of Science and Technology (KNUST), Kumasi, Ghana; 3 Centre for Tropical Clinical Pharmacology & Therapeutics, University of Ghana Medical School, Korle-Bu Teaching Hospital, Accra, Ghana; 4 Center for Pharmaceutical Management, Management Sciences for Health, Arlington, Virginia, USA

## Abstract

**Objective:**

To assess the appropriateness of self-reported use of anti-malarial drugs prior to health facility attendance, and the management of malaria in two health facilities in Ghana.

**Method:**

A structured questionnaire was used to collect data from 500 respondents who were diagnosed clinically and/or parasitologically for malaria at Agogo Presbyterian Hospital and Suntreso Polyclinic, both in the Ashanti Region of Ghana. Collected information included previous use of anti-malarial drugs prior to attending the health facilities, types of drugs used, how the drugs were used, and the sources of the drugs. In addition, the anti-malarial therapy given and outcomes at the two health facilities were assessed.

**Results:**

Of the 500 patients interviewed, 17% had severe malaria, 8% had moderate to severe malaria and 75% had uncomplicated malaria. Forty three percent of the respondents had taken anti-malarial drugs within two weeks prior to hospital attendance. The most commonly used anti-malarials were chloroquine (76%), sulphadoxine-pyrimethamine (9%), herbal preparations (9%) and amodiaquine (6%). The sources of these medicines were licensed chemical sellers (50%), pharmacies (21%), neighbouring clinics (9%) or "other" sources (20%) including left-over medicines at home. One hundred and sixty three (77%) of the 213 patients who had used anti-malarial drugs prior to attending the health facilities, used the drugs inappropriately. At the health facilities, the anti-malarials were prescribed and used according to the national standard treatment guidelines with good outcomes.

**Conclusion:**

Prevalence of inappropriate use of anti-malarials in the community in Ghana is high. There is need for enhanced public health education on home-based management of malaria and training for workers in medicine supply outlets to ensure effective use of anti-malaria drugs in the country.

## Background

Malaria is a public health problem in more than 90 countries. Each year, between 300 and 500 million new cases are reported worldwide [1]. According to the Roll Back Malaria Campaign of the World Health Organization (WHO), 90 percent of the more than one million deaths worldwide caused by malaria every year take place in Africa, and malaria constitutes 10 percent of the continent's overall disease burden [1,2]. In Africa south of the Sahara, malaria accounts for approximately 15% of deaths in children under five years of age and most of these occur in rural areas which have poor access to health care services [2-4].

In Ghana, malaria is responsible for up to 40% of daily out-patient consultations at hospitals and clinics and over 23% of deaths in children under five years of age [5-7]. Malaria is also responsible for 38,000 deaths per annum and over 2,000 deaths in pregnant women, accounting for 9% of all deaths in pregnancy [5,6].

Treatment and prevention of malaria deaths exist if the condition is promptly diagnosed, treated and/or referred. Delay in seeking therapy, misuse of anti-malarial drugs, and resistance of malaria parasites to existing drugs frustrate measures to effectively manage the disease in Africa [4,5,7-10].

In Ghana, most malaria cases are managed at the household level. Help is usually sought from the community pharmacist or licensed chemical seller (registered suppliers of specified over-the-counter-medicines) after the initial therapy has failed. Most patients reporting at clinics and hospitals facilities would have gone through home-based treatment or community drug shops/pharmacies initially. Thus mainly resistant, severe, or recurrent episodes are seen at clinics and hospitals. There is concern that community and household management of malaria is often inadequate, inappropriate and ineffective. The current study was undertaken to assess the effectiveness and quality of home based and health facility management of malaria in two communities in Ghana.

## Methods

### Setting

The study was conducted on a cohort of patients diagnosed with malaria from Agogo hospital (a 240-bed mission and referral hospital in the country side, that provides specialist services in paediatrics, surgery, obstetrics & gynaecology and ophthalmology) and Suntreso Polyclinic in Kumasi City (which has an average attendance of about 150 out-patients per day). Kumasi is the second largest city in Ghana.

Institutional approval for the study was obtained from medical authorities of the health facilities. Patient consent was sought from either patients or their caregivers in the case of children after the purpose of the study and guarantees of anonymity and their rights had been explained to them in either English or one of the local Ghanaian languages.

### Target population and sample

All patients attending the Suntreso polyclinic and Agogo Presbyterian hospital with a diagnosis/suspected diagnosis of malaria were targeted for inclusion in the study. The sample included 300 patients from Agogo and 200 from Suntreso polyclinic.

Data were collected consecutively on every other new case of malaria diagnosed either clinically and/or parasitologically. In addition, all in-patients and those who had been detained at the two facilities for further observation were included in the study. Sampling was done daily for a period of six weeks in April to May 2002.

Of the 500 patients included in the study, 407 were children under 12 years of age, and 93 were adult patients. Blood film examinations were used to diagnose 315 of the malaria cases (63%) while 185 (37%) were diagnosed clinically. Definitions of these diagnosis categories are given in Table 1.

**Table 1:** Definitions of terms used in the study

*Clinical diagnosis*	Diagnosis based on clinical assessment of the patient by a medical practitioner
*Laboratory diagnosis*	Diagnosis based on the presence of plasmodia parasites in the blood film of patients.
*Correct dosage*	Standard dosage of the anti-malaria drug recommended for complete treatment of the disease, based on mg/kg body weight or that recommended in the STGs*
*Wrong or inappropriate use*	Wrong dosage, wrong frequency and/or wrong duration of therapy
*Uncomplicated malaria*	Mild to moderate malaria without complications
*Moderate to severe malaria*	Malaria with symptoms leading to the patient being detained or admitted but without complications
*Severe malaria*	Malaria with complications and evidence of high parasitaemia
*Clinical cure*	Rapid resolution of signs and symptoms of malaria associated with improved well being of the patients
*Treatment failure*	Worsening of fever and other symptoms or death after initiation of therapy

### The Questionnaire

The data collection tool used consisted of sections on patient details, previous episodes of malaria two weeks prior to hospital or polyclinic attendance, drugs or herbs used for the prior treatment of malaria and its appropriateness in terms of dosage, frequency and duration of use, and current anti-malarial medications given at the health facilities. In the case of the patients who had had previous episodes of malaria, the source of drugs or herbs used for the prior treatment was also recorded. In addition to the patient interviews, pill identification and evaluation of previous anti-malarial drug packages were used to assess the anti-malarial drug history prior to health facility attendance for malaria treatment. The questionnaire was then pre-tested on a sample of 20 patients (10 from each facility) as a pilot, before it was used for the study. Appropriateness of drug use (either by prescribers at health facilities or the patients before hospital attendance) was determined using recommended dosage regimen from the Ghana National Standard Treatment Guidelines 2000 (STGs).

This study was conducted nearly three years before Ghana's new malaria policy change was implemented in January 2005. At the time of the study, the national first line treatment for uncomplicated malaria was chloroquine monotherapy with amodiaquine and/or sulphadoxine-pyrimethamine being used as second line. Under the current policy, a combination of amodiaquine plus artesunate is the first line recommendation for uncomplicated malaria. Quinine was, and still is the recommended drug for severe malaria. Definitions of terms used in the study are presented in Table 1.

### Data analysis

The information was entered into a database by a single data entry clerk using SPSS, Version 11. Discrepancies were resolved by cross checking with the hard copy of the data collection instruments. Data analyses were performed using the same software.

## Results

### Use of antimalarials and patient-reported knowledge of malaria

Of the 500 patients interviewed, a total of 85 (17%) had severe malaria, 38 (8%) were cases of moderate to severe malaria and 377 (75%) had uncomplicated malaria (Table 2). The majority of severe malaria cases (89%) were children aged five years or less, indicating the vulnerability of this group to severe or complicated malaria.

**Table 2:** Severity of malaria according to age of participants

**Severity of malaria**	**Age of patient**		
	**5 years or less** **n (%)**	**Over 5 years** **n (%)**	**Total** **n (%)**
Uncomplicated	211 (67)	166 (90)	377 (75)
Moderate to severe	29 (9)	9 (5)	38 (8)
Severe	76 (24)	9 (5)	85 (17)
**Total**	**316 (100)**	**184 (100)**	**500 (100)**

Two hundred and thirteen (43%) of the patients had taken an anti-malarial drug within two weeks prior to attending the hospital or polyclinic. Of these, one hundred and sixty-two (76%) had used chloroquine, 19 (9%) sulphadoxine/pyrimethamine, 13 (6%) amodiaquine while 19 (9%) had used a variety of herbal preparations. One hundred and seven (50%) obtained their medications from licensed chemical sellers and 45 (21%) obtained their drugs from pharmacies. Nineteen (9%) had been treated at a health facility (neighbouring clinic) before and 42 (20%) used leftover drugs from their homes, relatives or friends.

Altogether, 163 patients (77 %) did not know how to use and/or had used the medicines inappropriately. Ninety-two (86%) of the 107 who obtained their medications from licensed chemical sellers used the medicines inappropriately while twenty four (53%) of the 45 patients who sourced anti-malarial drugs from pharmacies used the drugs inappropriately. Seven (37%) of the 19 patients who received their medicines from neighbouring clinics used the medicines inappropriately. All but two of the 42 patients (95%) who used left-over medicines from their homes and from friends or relatives used the medicines inappropriately (Table 3).

**Table 3:** Sources of anti-malaria drugs and the percentage of inappropriate use

**Source of antimalaria drug**	**Inappropriate use**		
	**n (%)**	**n %**	**95% CI**
Licensed Chemical Shop	107 (50)	92 (86)	79 – 93
Pharmacy	45 (21)	24 (54)	39 – 69
Other sources at home	42 (20)	40 (95)	88 – 100
Neighbouring clinics	19 (9)	7 (37)	15 – 59
**Total**	**213 (100)**	**163 (77)**	

An assessment of respondents' knowledge of what malaria was indicated some knowledge of uncomplicated malaria. All study participants associated mosquito bites and fever with malaria but only 24 % of participants knew the danger signs of malaria and when to refer. The participants could not identify the signs of complicated malaria. There was no significant difference in the knowledge of participants with low educational (junior secondary education and below) attainment and those with higher educational attainment in their knowledge of malaria.

### Facility-based malaria treatment

Out of the 500 study participants only 253 could be followed up to assess treatment outcomes. The rest could not be traced because they lived at distances too far from the facilities or the contact details they provided were incorrect or non existent. However, all the cases of severe and moderate to severe malaria were followed up because the patients were detained or admitted at the institutions.

Quinine, often used in combination with sulphadoxine/pyrimetahmine, was the preferred anti-malarial drug for severe malaria at Agogo Hospital. Fifty-one patients of the 85 with severe malaria were put on quinine with a 98% cure rate achieved (one patient died). Artemisinin derivatives were the preferred choice for severe malaria at Suntreso polyclinic. Artemisinin derivatives were prescribed for 34 patients with severe malaria. All of the patients on this regimen were successfully treated. Artesunate was used in 26 (80%) of the cases. None of the patients were put on a regimen of artemisin-based combination therapy. Of those 38 patients with moderate to severe malaria in both institutions, all of them received amodiaquine with a cure rate of 84%.

Chloroquine was prescribed in both institutions for 88 of the 130 patients with uncomplicated malaria who were followed-up. In these patients a clinical cure rate of 85% was achieved. Also in this group of patients with uncomplicated malaria, Sulphadoxine/pyrimethamine was used by 30 patients with a cure rate of 67%. In addition 12 patients used amodiaquine with a 100% clinical cure rate.

## Discussion

This study showed that inappropriate use of anti-malarial drugs was high among patients attending two major health facilities in the Ashanti region of Ghana. Inappropriate use was highest among those who used left-over drugs and those who obtained their drugs from licensed chemical sellers. Such inappropriate use could be due to poor training for medicines dispensers, limited public health campaigns on medicines use, or poor regulation of the pharmaceutical distribution system in the country. Interventions to improve the use of anti-malarial medicines in Ghana could include public campaigns on use of anti-malarial drugs among the general public, training health workers on the management and appropriate use of anti-malarial drugs, or more stringent regulation of licensed chemical sellers' shops. In Tanzania, licensing, regulation, and franchizing of chemical shops significantly reduced the inappropriate use of medicines [15]. Environmental sanitation, the use of insecticide-treated bed nets and other aspects of the Roll Back Malaria initiative [1,2,5,7,11], must also be properly and efficiently implemented.

The use of anti-malarial drugs by those who obtained them from pharmacies and clinics was more rational than by those who obtained their medicines from chemical sellers, and those who treated themselves with leftover medicines. Still, inappropriate use rates of 54% and 37% in pharmacies and clinics respectively are high and interventions to improve use of anti-malarial medicines should target these outlets as well.

In this study 35% of the cases were diagnosed clinically. In a previous study in Ghana, Dunyo and colleagues established that children diagnosed with malaria by caretakers in the household were as likely to have malaria parasitaemia as those diagnosed with malaria in health facilities [17]. This fact dismisses inaccurate diagnosis as the cause of inappropriate use of anti-malarial medicines in Ghana.

Quinine was the preferred anti-malarial drug for severe malaria at Agogo hospital, which is located in a rural area, about 85 km from Kumasi. Quinine has been and is still reserved for severe malaria treatment in Ghana, because it has retained its efficacy. This may be due to the fact that quinine is not easily available and routinely used like chloroquine and sulphadoxine/pyrimethamine in the homes and drug retail outlets in the communities in Ghana [12,14,18]. It is mostly available in the health facilities like hospitals and clinics, and is usually given as an injectable for severe malaria. In Agogo hospital, it was used in combination with sulphadoxine/pyrimethamine with good outcomes, rather than used as monotherapy as stated in the national treatment guidelines. Further investigation is required to ascertain whether the combination treatment is more effective than quinine alone. The artemisinin derivatives were preferred at the polyclinic in the city for management of severe malaria. This was also a deviation from the national treatment guidelines at that time, which recommended quinine. But most clinicians were more comfortable prescribing preparations that were easy to administer. The preference for artemisinin derivatives could also have been due to aggressive marketing of the products by pharmaceutical company representatives which was going on at the time of the study.

Treatment failure observed with common anti-malarial drugs in both institutions may be due to drug resistance. There is evidence that about 40% and 15% respectively of parasitological and clinical treatment failure associated with chloroquine in sub-Saharan Africa is due to drug resistance [12-14,18]. This study did not ascertain the precise reasons that accounted for the over 30% treatment failure to sulphadoxine/pyrimethamine observed. It could be due to quality of the drugs or drug resistance. Like chloroquine, there are also reports of resistance to sulphadoxine/pyrimethamine in sub-Saharan Africa [12,18].

The most commonly used anti-malarial drug reported for treating previous episodes of malaria before attending the health facilities was chloroquine. Chloroquine was the first line drug for uncomplicated malaria, and the recommended drug for home-base management of malaria in Ghana at the time of the study. Various published studies in the late 90s showed that appropriate use of chloroquine was associated with good patient outcomes, whereas inappropriate or inadequate use was associated with treatment failure as was observed in this study [1-3,8,11,13]. In most endemic countries in Africa, including Ghana, increasing reports of widespread resistance to chloroquine and other common antimalarial drugs has necessitated a change in policy to Artemisinin Combination Therapy (ACT) as first line treatment for uncomplicated malaria. In Ghana, the recommended ACT is artesunate-amodiaquine. This has huge economic implications for most poor patients in sub-Saharan Africa, since the ACTs are comparatively more expensive. There are several legitimate concerns about the sustainability of this policy but the change is necessary for effective management of malaria in Ghana, due to the widespread multi-drug resistant strains of malaria parasites in the West African sub-region and Ghana in particular. In addition to the financial burden to be incurred as a result of this change, it is important to ensure appropriate usage of these medicines to prevent development of resistance to the artemisinins and related combination products, and to reduce cost associated with treatment failures. A national health insurance scheme has been instituted in Ghana supported by an act of parliament that allows the public to access health care for diseases of public health concern without making an out-of-pocket payment for attending a health institution or paying for drug prescriptions. Effective implementation of this scheme may provide respite economically for use of ACT for uncomplicated malaria in Ghana.

Limitations of this study include the fact that 247 patients (49% of the study participants) were lost to follow-up for outcomes assessment at the health facilities. These were all outpatients whose addresses were not traceable. This in itself is not surprising considering the very high migration rate within the community studied and the nation at large. Furthermore, the lack of a proper address system in the study areas leads to huge inevitable loss of patients in such study designs which happen to be the most pragmatic. However, all the patients with moderate to severe and complicated malaria, were followed up for outcomes assessment in the health facilities. It is strongly recommended to health policy makers and local governments to put in place systems for effective follow up of patients treated at OPD units of health facilities in Ghana, for therapeutic outcomes evaluation. Another limitation is targeting only one region in the country and only two facilities within that region. The reported results may, therefore, not fully represent the country as a whole but most likely to serve as a fair estimate of the situation in Ghana.

## Conclusion

The study has shown that there is a high prevalence of anti-malarial drug usage in the community prior to hospital attendance and that in most cases these drugs are used inappropriately. Effective and pragmatic strategies must be adopted to enhance public health education on safe and effective use of anti-malaria drugs in the community. In particular it is strongly recommended that appropriate training be provided for staff in the pharmacies, licensed chemical shops and other dispensers in health care facilities in Ghana, on the need to advice clients or patients on adherence to appropriate treatment regimen for malaria and other interventions for malaria control to reduce morbidity and mortality.

## Authors' contributions

KOB, the corresponding author was the principal investigator for this project, played an active role in data collection, data analysis and drafting of the manuscript.

LM, ANOD and MD were all actively involved in the design of the project and contributed to the drafting and reviewing of the manuscript. HE was the Project supervisor, and was involved with the drafting and reviewing of the manuscript. All the authors read and approved the final manuscript.
